# Role of CT Coronary Angiography at Initial Presentation in Kawasaki Disease—Insights from a Tertiary Care Center in North India

**DOI:** 10.3390/diagnostics15141806

**Published:** 2025-07-17

**Authors:** Manphool Singhal, Rakesh Kumar Pilania, Suprit Basu, Dev Desai, Abarna Thangaraj, Ripudaman Singh, Radhika Semwal, Taranpreet Kaur, Gopika Sri, Murugan Sudhakar, Arun Sharma, Pandiarajan Vignesh, Deepti Suri, Surjit Singh

**Affiliations:** Postgraduate Institute of Medical Education and Research, Chandigarh 160012, India; basusuprit@gmail.com (S.B.); devcdesai@gmail.com (D.D.); dr.abarna.t@gmail.com (A.T.); singhripudaman57@gmail.com (R.S.); radhikasemwal23@gmail.com (R.S.); taranpreetkaur2196@gmail.com (T.K.); gopikasri1711@gmail.com (G.S.); drmurugansudhakar20@gmail.com (M.S.); drarungautam@gmail.com (A.S.); vigimmc@gmail.com (P.V.); surideepti@gmail.com (D.S.); surjitsinghapc@gmail.com (S.S.)

**Keywords:** CT coronary angiography, coronary artery abnormality, Kawasaki disease, acute phase, missed aneurysm

## Abstract

**Background**: Kawasaki disease (KD) is a systemic vasculitis and the leading cause of acquired heart disease in children. Early identification of coronary artery abnormalities (CAAs) is crucial to guide treatment and improve outcomes. While transthoracic 2D echocardiography (TTE) remains the first-line imaging modality, it has limitations, particularly in visualizing distal coronary artery segments and detecting thrombi. Computed tomography coronary angiography (CTCA) offers enhanced visualization, but its role at initial presentation of KD remains underexplored. **Methods**: We reviewed the records of 71 children with KD who underwent CTCA at their initial presentation at a tertiary center between November 2013 and December 2024. The CTCA findings were compared with those of TTE. CTCA was performed after stabilization using radiation-minimized protocols. **Results**: Of 71 patients, 62 had CAAs on baseline TTE. CTCA confirmed CAAs in 39 patients, identified additional lesions in 23, and detected distal aneurysms and coronary branch involvement missed by TTE. In 20 patients with initially abnormal TTE, CTCA demonstrated normal coronaries, facilitating treatment de-escalation. CTCA identified coronary thrombi missed on TTE in two patients and congenital coronary anomalies in three patients. CTCA findings led to modification of therapy in multiple cases. **Conclusions**: CTCA is a valuable adjunct to TTE in evaluating coronary artery involvement at the time of initial presentation of children with KD. Given its superior visualization of the entire length of coronary arteries, CTCA has a vital role in therapeutic decision-making in KD.

## 1. Introduction

Kawasaki disease (KD) is an acute systemic vasculitis of unknown etiology that predominantly affects children below 5 years. It is the most common cause of acquired heart disease in children [1,2,3]. Without treatment, one out of four children with KD can develop coronary artery abnormalities (CAAs), underscoring the critical need for early diagnosis and treatment to reduce this risk [3,4,5].

Cardiac imaging is crucial for assessing coronary involvement in KD. Transthoracic 2D echocardiogram (TTE) is the primary imaging modality for diagnosing coronary arteries in KD [6,7]. However, it has several limitations. These include observer dependency and inability to assess CAAs in distal segments of coronary arteries and the left circumflex coronary artery (LCx). TTE is also not the best imaging modality for detecting coronary thrombosis. Computed tomography coronary angiography (CTCA) has the potential to address many of these limitations of TTE [8,9].

The 2024 American Heart Association (AHA) scientific statement on diagnosing and managing KD recommends CTCA on follow-up for evaluating patients with KD who develop CAAs. It has been suggested that CTCA may be performed 1 year after diagnosis, and subsequently every 3–5 years [2]. However, there are currently no guidelines on the role of CTCA as an imaging modality at the time of initial presentation. There is a paucity of literature on this aspect of KD. Herein, we report our experience on the role of CTCA in the evaluation of coronary arteries in children with KD at initial presentation.

## 2. Methodology

We reviewed records of children with KD who underwent CTCA for coronary artery assessment at our center. Our institute is a federally funded, not-for-profit, tertiary care teaching institute in North India. The diagnosis of KD was based on the American Heart Association (AHA) scientific statement [2,3,10]. CTCA was performed on 376 patients between November 2013 and December 2024. Amongst these, patients’ records of those who underwent CTCA at the initial presentation were analyzed in detail. Patient demographics (age at diagnosis, gender, date of TTE, and date of CTCA) and findings of TTE and CTCA were entered on a predesigned proforma. CTCA was performed at the time of initial presentation after the child had stabilized. Informed written consent was obtained from all parents/guardians before subjecting the children to CTCA. Findings on CTCA were compared with TTE performed around that time and with baseline TTE. The cardiac radiologist (MS) was, however, blinded to the findings of TTE. The manuscript was approved by the Departmental Publication Review Board (No. RDG/EC/Pub/27). No funding was involved in this study.

CTCAs were performed on a 128-slice scanner (Siemens Somatom Definition Flash, Erlangen, Germany) during the period January 2013 to February 2022, and thereafter on a 192-detector dual-source CT scanner (Siemens Somatom Force, Erlangen, Germany). No sedation was necessary in children above the age of 5 years. For children below 5, oral triclofos (50 mg/kg/dose) was used as a single dose. In addition, some children required intravenous midazolam (0.1–0.2 mg/kg/dose).

Radiation exposure was optimized using an adaptive prospective electrocardiography-gated sequence, automated tube current modulation (Care Dose 4D; Siemens Healthineers, Erlangen, Germany), lower kilovoltage settings (fixed at 80 kVp), and iterative image reconstruction algorithms (Safire/Admire; Siemens Healthineers, Erlangen, Germany).

Echocardiography assessment and interpretation of CAAs: TTE was performed at admission, and repeated during the hospital course as mandated by clinical requirements. In most cases, it was also performed at discharge and, thereafter, 2–6 weeks on follow-up. TTE was carried out by postdoctoral fellows trained in cardiovascular assessment of KD under the supervision of faculty members. Before 2019, TTE was performed using an Esaote MyLab30Gold ultrasound machine (Esaote S.p.A., Genowa, Italy), and since then, it has been conducted on the Philips EPIQ 7G ultrasound system (US 51881625) (Philips Ultrasound, Inc., Bothell, WA, USA). In 2013, we began assessing coronary arteries in children with KD using Z-scores [11]. Before this, we evaluated CAAs using absolute diameters.

CTCA assessment and interpretation of CAAs: Post-processing analysis was carried out on a dedicated Syngovia (Siemens Healthineers) workstation to reconstruct and evaluate coronary arteries. All CTCAs were reviewed by a single, experienced cardiac radiologist (M.S.). Coronary arteries and side branches were included in the analysis wherever these were visualized. CAAs were evaluated in terms of location, number, and morphology (i.e., aneurysms—saccular/fusiform; and dilatation). The term ‘aneurysm’ was used if the internal diameter of the coronary artery was ≥1.5 times that of an adjacent segment. Coronary arteries were said to be ‘dilated’ if the internal diameter was increased, but was <1.5 times that of an adjacent segment [3,10]. CAAs on CTCA were recorded, and the results were compared with the findings on TTE, including both the baseline TTE and the one performed immediately prior to CTCA.

## 3. Results

A total of 71 patients with KD who underwent both TTE and CTCA at the time of initial presentation were identified through a review of records.

**(a)**  **Patient characteristics:**

Among the 71 patients, 50 (70.4%) were male. Median age was 39 months (2–360 months), and 22 (30.1%) were infants. All patients had fever at presentation, with a median duration of fever of 11 days (range 3–60 days). Median time to KD diagnosis was 15 days (4–60 days) from the onset of symptoms. Complete KD was diagnosed in 49 patients, and 22 had incomplete KD. KD shock syndrome was seen in eight (11.3%) patients, and macrophage activation syndrome in two (2.8%) patients, respectively. Laboratory investigations revealed a median hemoglobin value of 92.5 g/L (range 58–138), total leukocyte count 16.02 × 10^9^/L (range 3.22–53.20), platelet count 6.94 × 10^9^/L (range 230–1635), serum albumin 3.15 g/dL (range 1.2–6.4), and C-reactive protein 44.5 mg/L (range 0.1–279). All patients received treatment with intravenous immunoglobulin (IVIg) and aspirin. Treatment augmentation was required in 54 patients. This was carried out with infliximab in 46 patients (64.8%) and steroids in 31 (43.7%) patients, respectively (Table 1).

**(b)**  **Indications of CTCA:**

**Figure 1:**
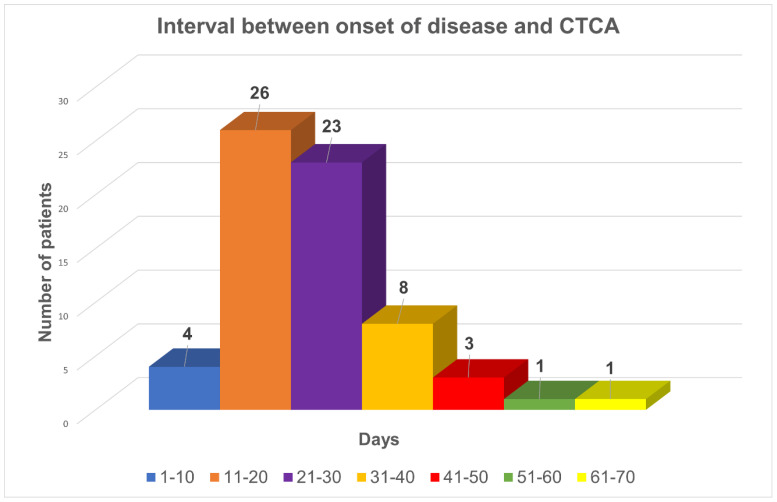
Distribution of patients by interval (in days) between the onset of disease and CTCA.

**Figure 2:**
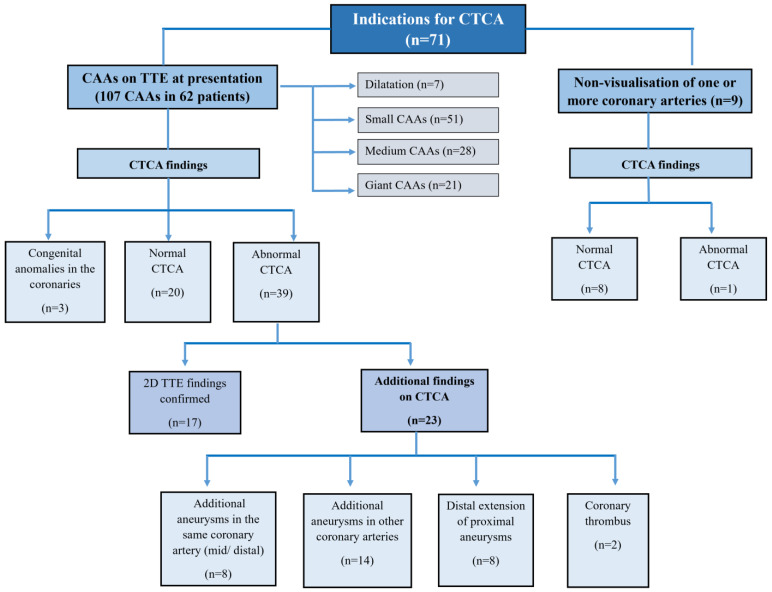
Indications for CTCA in our cohort and correlation between CTCA and TTE findings.

Median interval between disease onset and the performance of CTCA was 23 days (range: 9–85 days) (Figure 1). Indications for CTCA were: (i.) CAAs (aneurysms/dilatations/non-tapering of coronary arteries) on TTE at presentation (*n* = 62); (ii.) non-visualization of one or more coronaries on TTE (*n* = 8); and (iii.) non-availability of an appropriate window to carry out TTE (*n* = 1) (Figure 2).

**(c)**  **Findings on TTE and CTCA**

**Figure 3:**
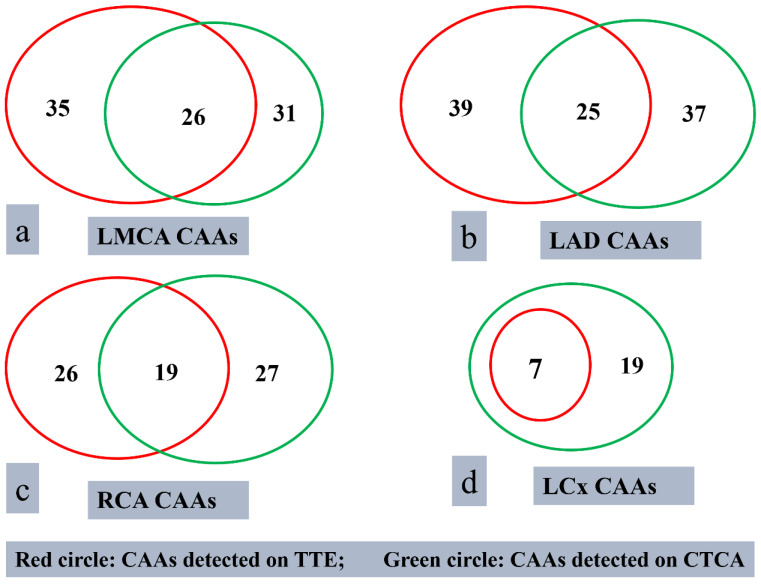
Comparison of CAAs detected on TTE (red circle) and CTCA (green circle).

- Imaging by TTE:

TTE identified 107 CAAs in 62 patients (Figure 3). Amongst these, 42 patients had CAAs detected on both baseline TTE and the TTE performed at the time of CTCA. In the remaining 20 patients, CAAs were observed on baseline TTE, but subsequent TTE at the time of CTCA showed normalization of coronary artery findings. In eight patients, one or more coronaries could not be assessed on TTE. In one patient, TTE could not be performed because of an infectious skin lesion over the anterior chest wall. On TTE, the left anterior descending (LAD) artery was the most commonly affected (*n* = 39), followed by the left main coronary artery (LMCA) (*n* = 35), right coronary artery (RCA) (*n* = 26), and left circumflex artery (LCx) (*n* = 7) (Figure 2).

ii. Imaging by CTCA:

Of the 62 patients with CAAs detected on TTE, CTCA confirmed the presence of CAAs in 39 patients, while 20 had normal coronary arteries. These 20 patients also showed normal findings on TTE performed at the time of CTCA. In three patients, congenital coronary anomalies were later identified, which had been misinterpreted as CAAs on the initial TTE [12].

iii. CAAs detected, or confirmed, on CTCA

**Figure 4:**
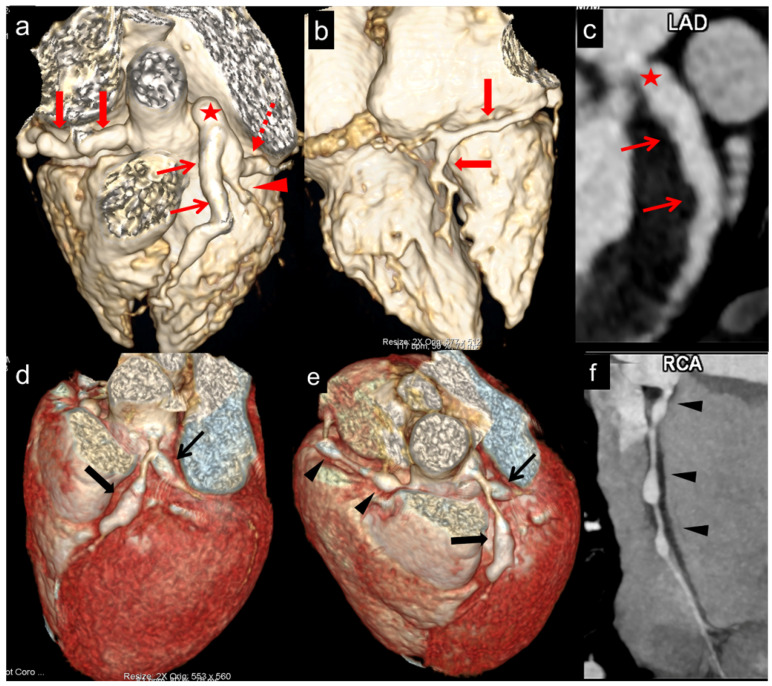
(**a**–**c**) (Patient No.3 in Table 2): CT coronary angiography (CTCA) volume rendered (**a**,**b**) and curved reformatted of left anterior descending (LAD) (**c**) images in a 7 months male infant at presentation show fusiform aneurysm of right coronary artery (RCA) along its entire course with extension into posterior descending branch (PDA) (thick arrows in **a**,**b**). The left main coronary (LMCA) (asterisk in **a**,**c**), proximal and mid anterior descending (LAD) (thin arrows in **a**,**c**), and proximal left circumflex (LCX) (interrupted arrows in **a**) show fusiform aneurysms. Fusiform aneurysm is extending to involve the proximal segment of the obtuse marginal (OM) branch of LCX (arrowhead in **a**). Although TTE showed aneurysms in LMCA, proximal LAD, LCX, and RCA, it failed to demonstrate the distal extension of the aneurysm of RCA and involvement of the OM branch of LCX. (**d**–**f**) (Patient No.15 in Table 2): CTCA volume rendered (**a**,**b**) and curved reformatted images of RCA (**c**) in a 76 months boy at presentation show fusiform aneurysms of proximal left anterior descending (LAD) (thick arrows in **a**,**b**) and left circumflex (LCX) (thin arrows in **d**,**e**). RCA shows three discontinuous fusiform aneurysms in the proximal and mid segments (arrowheads in **e**,**f**). Although TTE showed aneurysms in proximal LAD, LCX, and RCA, it failed to demonstrate aneurysms in mid RCA.

**Figure 5:**
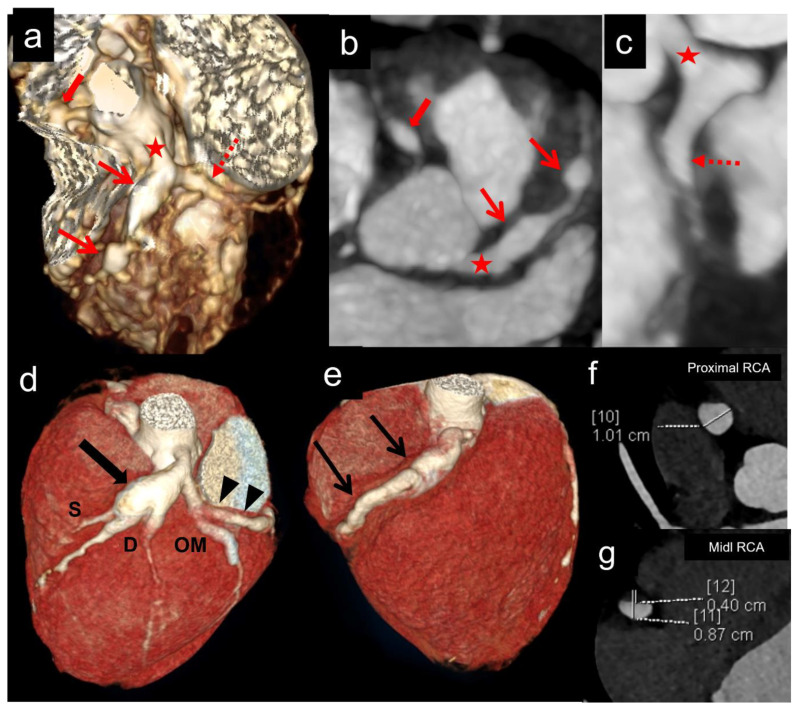
(**a**–**c**): (Patient No.12 in Table 2): CT coronary angiography (CTCA) volume rendered (**a**), axial (**b**) and curved reformatted images of left circumflex (LCX) (**c**) in a 2 months female infant at presentation show fusiform aneurysm of left main coronary artery (LMCA) (asterisk in **a**–**c**) with extension into osteo-proximal segments of LAD (thin arrow in **a**,**b**) and LCX (interrupted arrow in **a**,**c**). Another non-contiguous aneurysm is seen in mid LAD (second thin arrow in **a**,**b**). Although TTE showed aneurysms in LMCA, proximal LAD, and RCA, it failed to demonstrate a second non-contiguous aneurysm in mid LAD and a fusiform aneurysm in LCX. (**d**–**g**): (Patient No.22 in Table 2): CTCA volume rendered (**d**,**e**), axial proximal RCA (**f**), and distal RCA (**g**) images in a 96 months boy at presentation show fusiform aneurysm of LMCA with extension into osteo-proximal segments of LAD (thick arrow in d) and LCX (arrow heads in **d**). Note the extension/involvement of septal (S) and diagonal (D) branches of LAD and the obtuse marginal branch (OM) of LCX (**a**). RCA shows a fusiform aneurysm in its entire course (thin arrows in **e**). Axial images of RCA (**f**,**g**) show dilated RCA with eccentric thrombus in mid RCA (**g**). TTE showed aneurysms in LMCA, LAD, and RCA; however, it failed to demonstrate LCX aneurysm, distal extension of RCA aneurysm, and extension/involvement of septal (S) and diagonal (D) branches of LAD and obtuse marginal branch (OM) of LCX. The thrombus in the mid RCA aneurysm was also missed.

CTCA detected a total of 114 CAAs in 39 patients. These were seen in LAD (*n* = 37), LMCA (*n* = 31), RCA (*n* = 27), and LCx (*n* = 19) (Figure 3). CTCA detected six aneurysms in the distal segments of the coronary arteries—these had been missed on TTE. Among the 39 patients with CAAs detected on CTCA, 23 had additional findings missed on TTE (Figure 2). CTCA also identified additional aneurysms in the same coronary artery (*n* = 8) and new aneurysms in another coronary artery (*n* = 14) that had been missed on TTE (Figure 4). There was distal extension of the proximal CAAs in 12 patients. At the same time, involvement of coronary artery branches was noted in nine (Figure 4 and Figure 5)—these findings had been missed on TTE and demonstrated on CTCA (Table 2).

In one patient suspected of having a thrombus on TTE, imaging by CTCA revealed normal findings. This finding facilitated the discontinuation of anticoagulation therapy. In two patients, three thrombi in CAAs were diagnosed on CTCA. Of these three thrombi, one had been detected on TTE, while two had been missed. Two thrombi were in RCA (Figure 5), whereas one was in LAD. Both the thrombi missed on TTE were seen in the RCA.

iv. Patients with congenital coronary artery anomalies on CTCA

CTCA identified incidental congenital coronary artery anomalies in three children, but on TTE, these had been misinterpreted as CAAs. One patient had an anomalous left coronary artery from the pulmonary artery (ALCAPA), and TTE showed a dilated RCA. In the remaining two patients, one had separate origins of the left LAD and LCx from the left sinus with an absent left main trunk, while the other had a single coronary artery. Both had been misinterpreted as dilated LMCA on TTE [12].

v. Patients with no CAAs on CTCA

CTCA demonstrated normal coronary arteries in 20 patients. In all of these cases, baseline TTE had shown CAAs; however, TTE performed on the day of CTCA revealed normal coronary findings. The median interval between the initial TTE and CTCA was 72 h (24 h to 50 days) (Figure 1). Among these 20 patients, 15 had CAAs involving a single coronary artery, while 5 had involvement of two coronary arteries. All aneurysms detected in the LMCA (*n* = 10) and RCA (*n* = 7) were small. In the LAD, eleven aneurysms were small, while three were medium-sized. The time intervals between the first TTE and CTCA in patients with medium-sized LAD aneurysms were 21 days, 40 days, and 50 days, respectively.

**(d)**  **Impact of CTCA on management:**

Aspirin treatment was discontinued in three patients with congenital coronary anomalies once CTCA ruled out the presence of CAAs. In one patient with an infective skin lesion over the anterior chest wall, TTE could not be performed, and CTCA was carried out. As this showed no abnormality, aspirin could be discontinued. Moreover, CTCA provided critical additional information in two patients where TTE had failed to detect coronary thrombi. CTCA revealed previously unrecognized intraluminal thrombi within the coronary arteries in these patients. This led to a change in management strategy—low molecular weight heparin (LMWH) was introduced as an anticoagulant. In both cases, this was accompanied by intensified immunomodulatory therapy, which included IVIg, infliximab, corticosteroids, and cyclosporine.

## 4. Discussion

The most important complication of KD is the development of CAAs [9]. Although CAAs have been reported to occur in the proximal segments of coronary arteries, these can also occur in distal segments and branches of coronary arteries [13,14]. This study compares the diagnostic performance of TTE and CTCA in children with KD at the time of initial presentation. Our findings suggest that CTCA provides explicit visualization of coronary arteries along their entire length. This helps detect CAAs in distal segments of coronary arteries that may be missed on TTE. The role of CTCA at the time of initial presentation of KD is particularly valuable, as early identification of coronary involvement enables appropriate risk stratification and timely intervention. As the highest risk of developing coronary aneurysms occurs within the first two weeks of illness, a state-of-the-art imaging modality like CTCA can significantly impact treatment decisions. Unlike TTE, which has limited visualization of distal coronary artery segments, CTCA facilitates comprehensive 3D imaging. This provides a more detailed assessment of aneurysm morphology, distal extension of proximal aneurysms, non-contiguous aneurysms in distal segments, involvement of coronary branch arteries, and coronary thrombus [8,9,15].

In 20 patients, where baseline TTE was abnormal (small aneurysms (*n* = 17); medium aneurysms (*n* = 3)), CTCA showed normal coronaries. A normal CTCA study facilitated the discontinuation of aspirin at 6 weeks. Furthermore, a regular CTCA examination helped de-escalate the therapeutic regimens, reducing unnecessary medication exposure and obviating the need for follow-up CTCA [16]. Additionally, normal CTCA findings can mitigate unnecessary anxiety and concern for patients and families. All patients in this cohort with a normal CTCA have remained well on follow-up. In our study, CTCA identified aneurysms in 23 patients that had been missed or underestimated by TTE. In nine patients, CTCA was performed due to the inability of TTE to visualize the coronary arteries adequately. CTCA helped optimize therapeutic decisions in these patients, such as stopping anticoagulation therapy in patients where CTCA ruled out a suspected thrombus on TTE. CTCA also helped initiate anticoagulation therapy in two patients, wherein a thrombus had been missed on TTE. These findings highlight the potential of CTCA to guide both treatment escalation (e.g., immunomodulatory therapy, anticoagulation) and de-escalation decisions in children with KD based on a more accurate assessment of coronary artery involvement [2].

The superiority of CTCA over TTE in detecting CAAs in KD has been well documented [14,17,18,19]. We have previously shown that CTCA is particularly useful in evaluating distal coronary artery segments, which are often difficult to visualize on TTE because of inappropriate acoustic windows [14]. In the present study, CTCA revealed six additional distal aneurysms missed on TTE. Similarly, CTCA identified more aneurysms in the LCx artery (*n* = 19) than TTE (*n* = 7). In patients with suspected coronary thrombi, CTCA helped clarify the diagnoses and facilitated therapeutic decision-making. These findings reinforce that CTCA enhances diagnostic accuracy, especially in patients with CAAs involving distal coronary arteries, LCx, or branches of coronary arteries, and also in patients with coronary thrombi. Additionally, TTE may miss congenital coronary anomalies, especially when these are not suspected clinically before TTE. Further, these are liable to be misdiagnosed as CAAs [12]. We contend that CTCA is a useful adjunct to TTE at the initial presentation of KD. While TTE is widely accessible and cost-effective, it may not be sufficient as the sole imaging modality in KD [14,17,20].

The 2024 AHA scientific statement recommended serial CTCAs on follow-up in KD patients with CAAs at presentation. However, its use during initial presentation has not been explicitly addressed [2]. Based on our findings and experience, we propose that CTCA be integrated into the standard evaluation protocol of coronary arteries in children with KD at the time of initial presentation, in selected cases. These include cases where CAAs have been detected on 2DE, older children and adolescents with poor acoustic windows for 2DE, children with a severe disease course (e.g., KD shock syndrome, KD with macrophage activation syndrome, or symptomatic myocarditis), infants under 6 months of age, and children with equivocal 2DE findings [9]. By providing comprehensive coronary artery visualization, CTCA offers critical information for risk stratification, treatment planning, and long-term cardiovascular monitoring.

The strengths of our study include our center’s more than 30 years of clinical experience in managing children with KD. The diagnosis and treatment of KD were based on standard guidelines, with the senior author (SS) personally managing this cohort. Additionally, the study utilized a standardized imaging protocol and evaluated CTCA in a large cohort of KD patients at the time of initial presentation. All CTCAs were performed under the direct supervision of a senior cardiac radiologist (MS). To the best of our knowledge, this is the first study to examine the role of CTCA in children with KD at initial presentation. The retrospective nature of the research and a single-center setting are limitations that may affect generalizability. Additionally, while radiation exposure from CTCA was minimized using radiation-optimized protocols, it remains a consideration in children. Importantly, no z-score systems are currently available for CTCA in children; hence, absolute coronary dimensions were used. Applying TTE-based z-scores to CTCA coronary diameters may not be appropriate, especially for the mid/distal segments of coronary arteries. Furthermore, our cohort’s median time to diagnosis was 11 days (range: 3–60 days), reflecting potential diagnostic delays commonly encountered in low- and middle-income countries. Larger and multicentric prospective studies are needed to validate our findings and assess the long-term impact of early CTCA assessment on clinical outcomes of KD. Additionally, advancements in CT imaging, such as ultra-low-dose protocols and artificial intelligence-assisted analysis, may further enhance its safety and diagnostic yield in children with KD. It is important to note that CTCA was primarily performed based on abnormal or inconclusive echocardiographic findings. In cases with incomplete visualization on echocardiography, CTCA identified significant abnormalities, demonstrating its added diagnostic value. We propose that evolving clinical indications may lead to broader use of CTCA, particularly in selected cases during the acute phase of KD.

## 5. Conclusions

We have shown the utility of CTCA as a valuable adjunct to TTE in evaluating coronary artery involvement at the time of initial presentation of children with KD. Given its superior visualization of the entire length of coronary arteries, CTCA has a vital role in therapeutic decision-making in KD. This can facilitate earlier risk stratification and adoption of more precise treatment strategies. Future updates to KD clinical guidelines should consider incorporating CTCA during initial presentation in the management algorithm, especially in cases where TTE findings are inconclusive.

## Figures and Tables

**Table 1:** Clinical details, laboratory profile, and treatment of the patients in our cohort.

Characteristic	*N* = 71
**Demographics**	
Age at presentation (median, range)	39 months (2–360)
Male sex	50 (70.4%)
**Clinical characteristics**	
Duration of fever (median, range)	11 days (3–60)
Delay in diagnosis (median, range)	15 days (4–60)
Interval between onset of disease and CTCA (median, range)	23 days (9–85)
Fever	71 (100%)
Rash	57 (80.3%)
Mucosal changes	57 (80.3%)
Extremity changes	27 (38%)
Eye changes	39 (54.9%)
Cervical lymphadenopathy	21 (29.6%)
Complete KD	49 (69%)
Peeling	9 (12.7%)
Beau’s lines	13 (18.3%)
**Laboratory features**	
Hemoglobin, g/L (median, range)	92.5 (58–138)
Total leukocyte count, cells/mm^3^ (median, range)	16,015 (3220–53,020)
Neutrophil, % (median, range)	57% (13–98)
Platelet count, cells/mm^3^ (median, range)	694,000 (230,000–1,635,000)
AST, U/L (median, range)	41.0 (12–1236)
ALT, U/L (median, range)	30.5 (6–428)
Albumin, g/dL (median, range)	3.15 (1.2–6.4)
Serum sodium, mmol/L (median, range)	134.0 (123–140)
ESR, mm in 1st hr (median, range)	42.0 (2–115)
CRP, mg/L (median, range)	44.5 (0.1–279)
**Treatment**	
Standard of care	
IVIg	71 (100%)
Aspirin	71 (100%)
Treatment intensification	
Infliximab	46 (64.8%)
Steroids	31 (43.7%)
Repeat IVIg	7 (9.9%)
Cyclosporine	21 (29.6%)
Anti-coagulation	
LMWH	28 (39.4%)
Warfarin	4 (5.6%)

**Table 2:** Details of coronary artery abnormalities on TTE and CTCA in the subgroup of patients who had additional findings on CTCA.

S.no.	Age (Months)/Sex	TTE Findings (Measurement in mm/Z-Score)				Interval Between Diagnosis and CTCA (Days)	CTCA Findings				Detailed Description of Findings on CTCA
		LMCA	LAD	RCA	LCx		LMCA (mm)	LAD (mm)	RCA (mm)	LCx (mm)	
1	8/M	4.8 (8.36)	7 (26.24)	8.3 (23.52)	NV	15	8	7	6.4		Fusiform and saccular aneurysms in proximal and distal RCA, respectively. Saccular aneurysm involving distal LCA, incorporating/involving the origin of proximal LAD and LCX. No e/o thrombosis
2	55/F	2.9 (1.8)	2.5 (2.2)	2.6 (1.7)	NV	18	Normal	2.4	Normal	Normal	Segmental areas of dilatations in the proximal and mid LAD
3	7/M	5 (10.3)	4.7 (11.3)	4.2 (8.50)	1.7 (1.7)	19	7.9	3.5	4.6	3.2	Fusiform aneurysm involving all the coronary arteries; entire course of RCA with extension into PDA; LMCA, proximal and mid LAD, and proximal LCX with involvement of OM1 branch
4	39/M	3.3 (2.9)	5.1 (10.5)	0.8 (−2.83)	0.8 (−2.11)	16	5.2	5.4	3		Saccular aneurysm in mid LAD; areas of dilatations in RCA, LCA, proximal LAD
5	20/M	2.4 (2.03)	2.9 (4.98)	1.5 (−0.49)	1.2 (−0.12)	12	2.7	2.7	1.9	1.5	Dilated distal LCA and proximal LAD
6	64/M	3.1 (1.6)	1.7 −0.5	1.4 (−1.80)	1 (−1.98)	24	2.6	1.8	1.4	1.5	Dilated mild LAD
7	156/M	6.17 (7.42)	4.68 (6)	7.5 (10.63)	NV	41	6.3	5	6.6	4.6	Fusiform aneurysms in all coronaries: LMCA, proximal and mid RCA, LAD, and LCX.
8	132/M	2.89 (−0.37)	3.9 (3.62)	2.14 (−1.26)	NV	38	3.8	2.7	1.8	2.7	Dilated all coronary arteries: Diffuse LMCA and LAD; proximal and mid segments of RCA and proximal LCX
9	42/M	3.1 (3.85)	2.7 (2.5)	3.85 (6.43)	NV	14	3	3.2	4.4	2.7	Fusiform aneurysms in all coronaries: LMCA, entire RCA, including PDA and PLV branches, and proximal LAD and LCX.
10	43/M	5.2 (7.6)	3.2 (4.1)	3.2 (3.00)	NV	10	7	3.5	2.4	2.4	Fusiform aneurysm in LMCA with extension into origins of LAD and LCX. Dilated the entire RCA and LCX.
11	14/F	5.2 (9.93)	3.6 (4.03)	3.3 (2.76)	1.6 (2.76)	26	6.4	3.6	1	2	LMCA saccular aneurysm, which is extending into the proximal LAD and proximal LCX
12	2/F	2.6 (4.41)	3.1 (7.53)	2.8 (5.75)	NV	24	2.4	3.8	2.7	2.5	Fusiform aneurysm of LMCA with extension into osteo-proximal segments of LAD and LCX; another non-contiguous aneurysm was seen in mid-LAD LAD
13	204/M	3.2 (−0.33)	2.96 (0.84)	2.7 (−0.62)	NV	28	3.2	3.1	2.3	Normal	Dilated LMCA and proximal LAD
14	7/M	2.97 (5.33)	3.2 (5.91)	4.9 (6.88)	NV	19	2.7	4.2	5.8	3.3	Fusiform aneurysms in RCA (2 in number). Fusiform aneurysms in LCx (2 in number)
15	76/M	3 (1.13)	6.68 (12.33)	6.5 (9.89)	2 (0.22)	58	1.8	6.4	5.1	12.6 × 4.1	(1) Fusiform aneurysm 6.4 mm W ∗ 18 mm L in proximal LAD (2) Three discontinuous fusiform aneurysms in proximal and mid RCA 6.5 mm W ∗ 5.1 mm L, 10.6 mm W ∗ 6.3 mm L, 9.2 mm W ∗ 3.4 mm L (3) Fusiform aneurysms in LCx just beyond origin, 12.6 mm L × 4.1 mm W
16	44/M	4.5 (6.4)	4.2 (7.35)	4.2 (6.06)	2.3 (1.97)	33	Normal	5	8.2	4	Fusiform aneurysm in proximal RCA (8.2 mm W ∗ 31 mm L giant aneurysm), LAD (5 mm W ∗ 25 mm L), LCx (4 mm W ∗ 25 mm L). No e/o thrombus
17	132/M	4.2 (3.15)	3.6 (3.5)	2 (−1.13)	1.5 (−1.36)	32	5.8	9.1	12.6	6.1	Multiple aneurysms in the entire course of RCA, with a giant aneurysm in its proximal segment; fusiform giant aneurysm in proximal LAD; fusiform aneurysm in proximal LCx; and a dilated LMCA
18	60/M	3.5 (2.23)	6.8 (16.05)	5.7 (10.04)	NV	26	3.6	7.7	6	3.1	LMCA normal; proximal LAD: fusiform aneurysm (7.7 mm W ∗ 50 mm L), with involvement of diagonal branch (3.6 mm), mid-segment: skip areas of dilatation. Distal segment: dilated (3.6 mm). RCA proximal segment dilated (6.6 mm for a length of 40 mm). AM branch: multiple skip aneurysms. LCx: proximal segment fusiform aneurysm (3.6 mm) with extension into OM branch (3.9 mm)
19	7/F	2.1 (2.06)	1.8 (0.65)	1.8 (3.30)	1.5 (0.16)	27	2.3	1.6	2.2	1.5	Dilated proximal and distal LAD with normal intervening segment
20	27/M	3.2 (2.95)	3.5 (5.42)	2.6 (1.98)		13	3.5	5.1		3.6	Fusiform aneurysm of LMCA extending to proximal LAD and LCx. No e/o thrombus
21	6/M	3.1 (4.6)	6.7 (16.82)	9.4 (24.00)	NV	35	3.8	6.6	8	4.1	Fusiform aneurysmal dilatation of all coronary arteries, no thrombus
22	96/M	4.85 (0.63)	8 (34)	7.5 (14.3)	NV	85	4.5	13	5.1	10	Fusiform aneurysm LMCA with extension into osteo-proximal segments of LAD and LCX and involving septal and diagonal branches of LAD and obtuse marginal branch of LCX; fusiform aneurysm in the entire course of RCA with an eccentric thrombus in mid RCA
23	7/M	2.47 (3.46)	14.9 (47.2)	4.17 (9.50)	4.39 (11.48)	51	2	18.3	3.5	7.2	Giant aneurysm in proximal LAD with partial thrombus (detected on TTE), proximal RCA saccular aneurysm with thrombus (not detected on TTE), LCx fusiform aneurysm (not detected on TTE)

TTE—Transthoracic 2D echocardiogram; CTCA—Computed tomography coronary angiography; LMCA—left main coronary artery, RCA—right coronary artery; LAD—left anterior descending artery; LCX—left circumflex coronary artery; OM—obtuse marginal branch; NV—Not visualized.

## Data Availability

This article’s data are available in the manuscript and in our records. These data can be shared on request.
